# Supplementary material for: Exogenous putrescine attenuates the negative impact of drought stress by modulating physio-biochemical traits and gene expression in sugar beet (Beta vulgaris L.)
Source: PLoS One. 2022 Jan 7;17(1):e0262099. doi: 10.1371/journal.pone.0262099 (PMC8741020; doi:10.1371/journal.pone.0262099)
Supplement: S1 Fig — (DOCX) [file pone.0262099.s001.docx]

| **Fig. #** | **Mean** | **SD** | **Statistical method used** | **P value** | **# samples** |
| --- | --- | --- | --- | --- | --- |
| **Fig. 1A** |  |  | Two way ANOVA/ Tukey’s post-hoc multiple comparison test | *P ≤ 0.05 | 4 |
| Con_BSRI sugar beet 2 | 9.36 | 0.27 |  |  |  |
| Con_SBT-010 | 8.63 | 0.17 |  |  |  |
| Drought (BSRI sugar beet 2) | 8.35 | 0.32 |  |  |  |
| Drought (SBT-010) | 7.36 | 0.22 |  |  |  |
| D + 0.3 mM Put (BSRI sugar beet 2) | 8.82 | 0.16 |  |  |  |
| D + 0.3 mM Put (SBT-010) | 9.65 | 0.18 |  |  |  |
| D + 0.6 mM Put (BSRI sugar beet 2) | 8.04 | 0.18 |  |  |  |
| D + 0.6 mM Put (SBT-010) | 9.42 | 0.19 |  |  |  |
| D + 0.9 mM Put (BSRI sugar beet 2) | 7.83 | 0.17 |  |  |  |
| D + 0.9 mM Put (SBT-010) | 9.25 | 0.15 |  |  |  |
| **Fig. 1B** |  |  | Two way ANOVA/ Tukey’s post-hoc multiple comparison test | *P ≤ 0.05 | 4 |
| Con_BSRI sugar beet 2 | 3.58 | 0.10 |  |  |  |
| Con_SBT-010 | 3.41 | 0.09 |  |  |  |
| Drought (BSRI sugar beet 2) | 3.12 | 0.12 |  |  |  |
| Drought (SBT-010) | 2.79 | 0.10 |  |  |  |
| D + 0.3 mM Put (BSRI sugar beet 2) | 3.39 | 0.04 |  |  |  |
| D + 0.3 mM Put (SBT-010) | 4.42 | 0.10 |  |  |  |
| D + 0.6 mM Put (BSRI sugar beet 2) | 2.84 | 0.16 |  |  |  |
| D + 0.6 mM Put (SBT-010) | 3.98 | 0.10 |  |  |  |
| D + 0.9 mM Put (BSRI sugar beet 2) | 2.86 | 0.08 |  |  |  |
| D + 0.9 mM Put (SBT-010) | 3.83 | 0.09 |  |  |  |
| **Fig. 1C** |  |  | Two way ANOVA/ Tukey’s post-hoc multiple comparison test | *P ≤ 0.05 | 4 |
| Con_BSRI sugar beet 2 | 2.61 | 0.14 |  |  |  |
| Con_SBT-010 | 2.52 | 0.02 |  |  |  |
| Drought (BSRI sugar beet 2) | 2.68 | 0.14 |  |  |  |
| Drought (SBT-010) | 2.63 | 0.04 |  |  |  |
| D + 0.3 mM Put (BSRI sugar beet 2) | 2.60 | 0.08 |  |  |  |
| D + 0.3 mM Put (SBT-010) | 2.18 | 0.01 |  |  |  |
| D + 0.6 mM Put (BSRI sugar beet 2) | 2.83 | 0.12 |  |  |  |
| D + 0.6 mM Put (SBT-010) | 2.36 | 0.06 |  |  |  |
| D + 0.9 mM Put (BSRI sugar beet 2) | 2.74 | 0.10 |  |  |  |
| D + 0.9 mM Put (SBT-010) | 2.41 | 0.09 |  |  |  |
| **Fig. 1D** |  |  | Two way ANOVA/ Tukey’s post-hoc multiple comparison test | *P ≤ 0.05 | 4 |
| Con_BSRI sugar beet 2 | 2.48 | 0.09 |  |  |  |
| Con_SBT-010 | 2.06 | 0.08 |  |  |  |
| Drought (BSRI sugar beet 2) | 2.18 | 0.07 |  |  |  |
| Drought (SBT-010) | 2.05 | 0.07 |  |  |  |
| D + 0.3 mM Put (BSRI sugar beet 2) | 2.26 | 0.10 |  |  |  |
| D + 0.3 mM Put (SBT-010) | 2.08 | 0.08 |  |  |  |
| D + 0.6 mM Put (BSRI sugar beet 2) | 2.10 | 0.03 |  |  |  |
| D + 0.6 mM Put (SBT-010) | 2.18 | 0.02 |  |  |  |
| D + 0.9 mM Put (BSRI sugar beet 2) | 1.96 | 0.04 |  |  |  |
| D + 0.9 mM Put (SBT-010) | 2.14 | 0.08 |  |  |  |
