# Supplementary material for: Exogenous putrescine attenuates the negative impact of drought stress by modulating physio-biochemical traits and gene expression in sugar beet (Beta vulgaris L.)
Source: PLoS One. 2022 Jan 7;17(1):e0262099. doi: 10.1371/journal.pone.0262099 (PMC8741020; doi:10.1371/journal.pone.0262099)
Supplement: S2 Fig — (DOCX) [file pone.0262099.s002.docx]

| **Fig. #** | **Mean** | **SD** | **Statistical method used** | **P value** | **# samples** |
| --- | --- | --- | --- | --- | --- |
| **Fig. 2A** |  |  | Two way ANOVA/ Tukey’s post-hoc multiple comparison test | *P ≤ 0.05 | 6 |
| Con_BSRI sugar beet 2 | 3.51 | 0.87 |  |  |  |
| Con_SBT-010 | 1.69 | 1.47 |  |  |  |
| Drought (BSRI sugar beet 2) | 1.19 | 0.92 |  |  |  |
| Drought (SBT-010) | 0.91 | 1.66 |  |  |  |
| D + 0.3 mM Put (BSRI sugar beet 2) | 1.92 | 0.23 |  |  |  |
| D + 0.3 mM Put (SBT-010) | 2.66 | 0.44 |  |  |  |
| D + 0.6 mM Put (BSRI sugar beet 2) | 2.43 | 0.59 |  |  |  |
| D + 0.6 mM Put (SBT-010) | 2.50 | 0.46 |  |  |  |
| D + 0.9 mM Put (BSRI sugar beet 2) | 2.11 | 0.54 |  |  |  |
| D + 0.9 mM Put (SBT-010) | 1.74 | 0.90 |  |  |  |
| **Fig. 2B** |  |  | Two way ANOVA/ Tukey’s post-hoc multiple comparison test | *P ≤ 0.05 | 6 |
| Con_BSRI sugar beet 2 | 1.64 | 0.28 |  |  |  |
| Con_SBT-010 | 0.42 | 0.23 |  |  |  |
| Drought (BSRI sugar beet 2) | 0.35 | 0.26 |  |  |  |
| Drought (SBT-010) | 0.38 | 0.27 |  |  |  |
| D + 0.3 mM Put (BSRI sugar beet 2) | 0.52 | 0.15 |  |  |  |
| D + 0.3 mM Put (SBT-010) | 0.60 | 0.13 |  |  |  |
| D + 0.6 mM Put (BSRI sugar beet 2) | 0.77 | 0.27 |  |  |  |
| D + 0.6 mM Put (SBT-010) | 0.60 | 0.10 |  |  |  |
| D + 0.9 mM Put (BSRI sugar beet 2) | 0.48 | 0.09 |  |  |  |
| D + 0.9 mM Put (SBT-010) | 0.49 | 0.38 |  |  |  |
| **Fig. 2C** |  |  | Two way ANOVA/ Tukey’s post-hoc multiple comparison test | *P ≤ 0.05 | 6 |
| Con_BSRI sugar beet 2 | 0.081 | 0.024 |  |  |  |
| Con_SBT-010 | 0.012 | 0.008 |  |  |  |
| Drought (BSRI sugar beet 2) | 0.009 | 0.008 |  |  |  |
| Drought (SBT-010) | 0.011 | 0.009 |  |  |  |
| D + 0.3 mM Put (BSRI sugar beet 2) | 0.017 | 0.005 |  |  |  |
| D + 0.3 mM Put (SBT-010) | 0.020 | 0.007 |  |  |  |
| D + 0.6 mM Put (BSRI sugar beet 2) | 0.024 | 0.010 |  |  |  |
| D + 0.6 mM Put (SBT-010) | 0.023 | 0.004 |  |  |  |
| D + 0.9 mM Put (BSRI sugar beet 2) | 0.014 | 0.003 |  |  |  |
| D + 0.9 mM Put (SBT-010) | 0.020 | 0.017 |  |  |  |
| **Fig. 2D** |  |  | Two way ANOVA/ Tukey’s post-hoc multiple comparison test | *P ≤ 0.05 | 6 |
| Con_BSRI sugar beet 2 | 2.31 | 0.55 |  |  |  |
| Con_SBT-010 | 3.22 | 2.86 |  |  |  |
| Drought (BSRI sugar beet 2) | 2.39 | 1.89 |  |  |  |
| Drought (SBT-010) | 1.57 | 0.92 |  |  |  |
| D + 0.3 mM Put (BSRI sugar beet 2) | 3.99 | 1.14 |  |  |  |
| D + 0.3 mM Put (SBT-010) | 4.64 | 0.65 |  |  |  |
| D + 0.6 mM Put (BSRI sugar beet 2) | 3.36 | 0.51 |  |  |  |
| D + 0.6 mM Put (SBT-010) | 4.08 | 1.50 |  |  |  |
| D + 0.9 mM Put (BSRI sugar beet 2) | 4.64 | 0.56 |  |  |  |
| D + 0.9 mM Put (SBT-010) | 4.47 | 1.15 |  |  |  |
