# Supplementary material for: Exogenous putrescine attenuates the negative impact of drought stress by modulating physio-biochemical traits and gene expression in sugar beet (Beta vulgaris L.)
Source: PLoS One. 2022 Jan 7;17(1):e0262099. doi: 10.1371/journal.pone.0262099 (PMC8741020; doi:10.1371/journal.pone.0262099)
Supplement: S3 Fig — (DOCX) [file pone.0262099.s003.docx]

| **Fig. #** | **Mean** | **SD** | **Statistical method used** | **P value** | **# samples** |
| --- | --- | --- | --- | --- | --- |
| **Fig. 3A** |  |  | Two way ANOVA/ Tukey’s post-hoc multiple comparison test | *P ≤ 0.05 | 4 |
| Con_BSRI sugar beet 2 | 2.89 | 0.44 |  |  |  |
| Con_SBT-010 | 2.45 | 0.32 |  |  |  |
| Drought (BSRI sugar beet 2) | 6.31 | 0.51 |  |  |  |
| Drought (SBT-010) | 4.64 | 0.29 |  |  |  |
| D + 0.3 mM Put (BSRI sugar beet 2) | 1.92 | 0.42 |  |  |  |
| D + 0.3 mM Put (SBT-010) | 2.63 | 0.24 |  |  |  |
| D + 0.6 mM Put (BSRI sugar beet 2) | 2.00 | 0.41 |  |  |  |
| D + 0.6 mM Put (SBT-010) | 1.54 | 0.33 |  |  |  |
| D + 0.9 mM Put (BSRI sugar beet 2) | 2.49 | 0.41 |  |  |  |
| D + 0.9 mM Put (SBT-010) | 2.02 | 0.32 |  |  |  |
| **Fig. 3B** |  |  | Two way ANOVA/ Tukey’s post-hoc multiple comparison test | *P ≤ 0.05 | 4 |
| Con_BSRI sugar beet 2 | 3.50 | 0.17 |  |  |  |
| Con_SBT-010 | 3.10 | 0.22 |  |  |  |
| Drought (BSRI sugar beet 2) | 4.45 | 0.32 |  |  |  |
| Drought (SBT-010) | 4.19 | 0.27 |  |  |  |
| D + 0.3 mM Put (BSRI sugar beet 2) | 2.76 | 0.16 |  |  |  |
| D + 0.3 mM Put (SBT-010) | 3.44 | 0.18 |  |  |  |
| D + 0.6 mM Put (BSRI sugar beet 2) | 3.80 | 0.11 |  |  |  |
| D + 0.6 mM Put (SBT-010) | 2.90 | 0.06 |  |  |  |
| D + 0.9 mM Put (BSRI sugar beet 2) | 3.41 | 0.19 |  |  |  |
| D + 0.9 mM Put (SBT-010) | 3.46 | 0.17 |  |  |  |
