# Supplementary material for: Exogenous putrescine attenuates the negative impact of drought stress by modulating physio-biochemical traits and gene expression in sugar beet (Beta vulgaris L.)
Source: PLoS One. 2022 Jan 7;17(1):e0262099. doi: 10.1371/journal.pone.0262099 (PMC8741020; doi:10.1371/journal.pone.0262099)
Supplement: S4 Fig — (DOCX) [file pone.0262099.s004.docx]

| **Fig. #** | **Mean** | **SD** | **Statistical method used** | **P value** | **# samples** |
| --- | --- | --- | --- | --- | --- |
| **Fig. 4A** |  |  | Two way ANOVA/ Tukey’s post-hoc multiple comparison test | *P ≤ 0.05 | 4 |
| Con_BSRI sugar beet 2 | 6.18 | 0.18 |  |  |  |
| Con_SBT-010 | 6.82 | 0.34 |  |  |  |
| Drought (BSRI sugar beet 2) | 5.13 | 0.04 |  |  |  |
| Drought (SBT-010) | 4.24 | 0.07 |  |  |  |
| D + 0.3 mM Put (BSRI sugar beet 2) | 6.67 | 0.09 |  |  |  |
| D + 0.3 mM Put (SBT-010) | 4.27 | 0.03 |  |  |  |
| D + 0.6 mM Put (BSRI sugar beet 2) | 6.27 | 0.03 |  |  |  |
| D + 0.6 mM Put (SBT-010) | 4.80 | 0.17 |  |  |  |
| D + 0.9 mM Put (BSRI sugar beet 2) | 4.74 | 0.08 |  |  |  |
| D + 0.9 mM Put (SBT-010) | 4.72 | 0.17 |  |  |  |
| **Fig. 4B** |  |  | Two way ANOVA/ Tukey’s post-hoc multiple comparison test | *P ≤ 0.05 | 4 |
| Con_BSRI sugar beet 2 | 4.78 | 0.29 |  |  |  |
| Con_SBT-010 | 6.39 | 1.08 |  |  |  |
| Drought (BSRI sugar beet 2) | 3.93 | 0.08 |  |  |  |
| Drought (SBT-010) | 1.23 | 0.04 |  |  |  |
| D + 0.3 mM Put (BSRI sugar beet 2) | 5.03 | 0.13 |  |  |  |
| D + 0.3 mM Put (SBT-010) | 3.60 | 0.60 |  |  |  |
| D + 0.6 mM Put (BSRI sugar beet 2) | 4.76 | 0.18 |  |  |  |
| D + 0.6 mM Put (SBT-010) | 2.81 | 0.52 |  |  |  |
| D + 0.9 mM Put (BSRI sugar beet 2) | 5.34 | 0.22 |  |  |  |
| D + 0.9 mM Put (SBT-010) | 2.74 | 0.52 |  |  |  |
| **Fig. 4C** |  |  | Two way ANOVA/ Tukey’s post-hoc multiple comparison test | *P ≤ 0.05 | 4 |
| Con_BSRI sugar beet 2 | 6.77 | 0.44 |  |  |  |
| Con_SBT-010 | 8.27 | 1.43 |  |  |  |
| Drought (BSRI sugar beet 2) | 5.87 | 1.68 |  |  |  |
| Drought (SBT-010) | 8.49 | 1.62 |  |  |  |
| D + 0.3 mM Put (BSRI sugar beet 2) | 3.37 | 0.96 |  |  |  |
| D + 0.3 mM Put (SBT-010) | 6.23 | 1.51 |  |  |  |
| D + 0.6 mM Put (BSRI sugar beet 2) | 6.69 | 2.20 |  |  |  |
| D + 0.6 mM Put (SBT-010) | 4.60 | 0.99 |  |  |  |
| D + 0.9 mM Put (BSRI sugar beet 2) | 5.50 | 1.46 |  |  |  |
| D + 0.9 mM Put (SBT-010) | 4.30 | 0.73 |  |  |  |
| **Fig. 4D** |  |  | Two way ANOVA/ Tukey’s post-hoc multiple comparison test | *P ≤ 0.05 | 4 |
| Con_BSRI sugar beet 2 | 1.44 | 0.21 |  |  |  |
| Con_SBT-010 | 4.92 | 0.72 |  |  |  |
| Drought (BSRI sugar beet 2) | 1.52 | 0.08 |  |  |  |
| Drought (SBT-010) | 2.92 | 0.43 |  |  |  |
| D + 0.3 mM Put (BSRI sugar beet 2) | 1.06 | 0.13 |  |  |  |
| D + 0.3 mM Put (SBT-010) | 2.86 | 0.40 |  |  |  |
| D + 0.6 mM Put (BSRI sugar beet 2) | 1.41 | 0.17 |  |  |  |
| D + 0.6 mM Put (SBT-010) | 3.20 | 0.58 |  |  |  |
| D + 0.9 mM Put (BSRI sugar beet 2) | 1.66 | 0.15 |  |  |  |
| D + 0.9 mM Put (SBT-010) | 2.33 | 0.36 |  |  |  |
