# Supplementary material for: Exogenous putrescine attenuates the negative impact of drought stress by modulating physio-biochemical traits and gene expression in sugar beet (Beta vulgaris L.)
Source: PLoS One. 2022 Jan 7;17(1):e0262099. doi: 10.1371/journal.pone.0262099 (PMC8741020; doi:10.1371/journal.pone.0262099)
Supplement: S5 Fig — (DOCX) [file pone.0262099.s005.docx]

| **Fig. #** | **Mean** | **SD** | **Statistical method used** | **P value** | **# samples** |
| --- | --- | --- | --- | --- | --- |
| **Fig. 5** |  |  |  |  |  |
| **Cu/Zn-SOD** |  |  | Two way ANOVA/ Tukey’s post-hoc multiple comparison test | *P ≤ 0.05 | 3 |
| Drought (BSRI sugar beet 2) | 1.098 | 1.32 |  |  |  |
| Drought (SBT-010) | -2.088 | 0.85 |  |  |  |
| D + 0.3 mM Put (BSRI sugar beet 2) | 2.239 | 0.94 |  |  |  |
| D + 0.3 mM Put (SBT-010) | -2.462 | 0.86 |  |  |  |
| D + 0.6 mM Put (BSRI sugar beet 2) | 4.868 | 2.85 |  |  |  |
| D + 0.6 mM Put (SBT-010) | -2.158 | 0.86 |  |  |  |
| D + 0.9 mM Put (BSRI sugar beet 2) | 2.112 | 0.37 |  |  |  |
| D + 0.9 mM Put (SBT-010) | -3.995 | 0.24 |  |  |  |
| **Fe-SOD** |  |  | Two way ANOVA/ Tukey’s post-hoc multiple comparison test | *P ≤ 0.05 | 3 |
| Drought (BSRI sugar beet 2) | -1.220 | 1.64 |  |  |  |
| Drought (SBT-010) | 0.996 | 0.38 |  |  |  |
| D + 0.3 mM Put (BSRI sugar beet 2) | 2.976 | 1.41 |  |  |  |
| D + 0.3 mM Put (SBT-010) | -0.221 | 0.09 |  |  |  |
| D + 0.6 mM Put (BSRI sugar beet 2) | 3.002 | 0.64 |  |  |  |
| D + 0.6 mM Put (SBT-010) | 0.014 | 0.70 |  |  |  |
| D + 0.9 mM Put (BSRI sugar beet 2) | -2.859 | 1.06 |  |  |  |
| D + 0.9 mM Put (SBT-010) | -0.723 | 0.29 |  |  |  |
| **Mn-SOD** |  |  | Two way ANOVA/ Tukey’s post-hoc multiple comparison test | *P ≤ 0.05 | 3 |
| Drought (BSRI sugar beet 2) | -0.929 | 0.58 |  |  |  |
| Drought (SBT-010) | 1.072 | 0.30 |  |  |  |
| D + 0.3 mM Put (BSRI sugar beet 2) | 2.833 | 1.33 |  |  |  |
| D + 0.3 mM Put (SBT-010) | 1.433 | 0.09 |  |  |  |
| D + 0.6 mM Put (BSRI sugar beet 2) | -1.656 | 1.93 |  |  |  |
| D + 0.6 mM Put (SBT-010) | 0.859 | 0.21 |  |  |  |
| D + 0.9 mM Put (BSRI sugar beet 2) | -1.401 | 2.32 |  |  |  |
| D + 0.9 mM Put (SBT-010) | 2.427 | 1.26 |  |  |  |
| **CAT** |  |  | Two way ANOVA/ Tukey’s post-hoc multiple comparison test | *P ≤ 0.05 | 3 |
| Drought (BSRI sugar beet 2) | 2.369 | 0.19 |  |  |  |
| Drought (SBT-010) | -2.482 | 0.55 |  |  |  |
| D + 0.3 mM Put (BSRI sugar beet 2) | 1.181 | 0.08 |  |  |  |
| D + 0.3 mM Put (SBT-010) | -1.932 | 0.73 |  |  |  |
| D + 0.6 mM Put (BSRI sugar beet 2) | 2.057 | 0.40 |  |  |  |
| D + 0.6 mM Put (SBT-010) | -2.478 | 0.65 |  |  |  |
| D + 0.9 mM Put (BSRI sugar beet 2) | 2.012 | 0.30 |  |  |  |
| D + 0.9 mM Put (SBT-010) | -3.084 | 0.32 |  |  |  |
| **APX** |  |  | Two way ANOVA/ Tukey’s post-hoc multiple comparison test | *P ≤ 0.05 | 3 |
| Drought (BSRI sugar beet 2) | 0.461 | 0.70 |  |  |  |
| Drought (SBT-010) | 0.891 | 1.78 |  |  |  |
| D + 0.3 mM Put (BSRI sugar beet 2) | 3.309 | 0.98 |  |  |  |
| D + 0.3 mM Put (SBT-010) | 4.768 | 1.32 |  |  |  |
| D + 0.6 mM Put (BSRI sugar beet 2) | 3.833 | 1.13 |  |  |  |
| D + 0.6 mM Put (SBT-010) | 0.166 | 1.27 |  |  |  |
| D + 0.9 mM Put (BSRI sugar beet 2) | -1.671 | 0.96 |  |  |  |
| D + 0.9 mM Put (SBT-010) | -0.998 | 0.66 |  |  |  |
