# Supplementary material for: Exogenous putrescine attenuates the negative impact of drought stress by modulating physio-biochemical traits and gene expression in sugar beet (Beta vulgaris L.)
Source: PLoS One. 2022 Jan 7;17(1):e0262099. doi: 10.1371/journal.pone.0262099 (PMC8741020; doi:10.1371/journal.pone.0262099)
Supplement: Attachment — Submitted filename: Resonse to reviewers.docx [file pone.0262099.s006.docx]

**Reviewer #1**: Authors have very well showed that application of putrescine (Put) alleviates drought stress in two sugar beet genotypes. Authors have studied effect of Put application in sugar beets during drought stress in various biological processes such as physiological, biochemical, and transcriptional level. Moreover, Authors have demonstrated that Put application enhances antioxidant levels thereby reduces H2O2 and MDA level during drought stress in Sugar beet genotypes.
However, there are several formatting errors and in detail explanation needed in material method section and in every figure legend. There are several grammatical errors in spacing, manuscript needs to be thoroughly checked for spacing errors.

Author’s response: Thanks for your constructive and meaningful comments regarding our MS that might help us for substantial improvement the MS. We have revised the MS based on your comments and other reviewer’s comments to make it more meaningful.

**Specific comments**

Why it has been not considered to treat control plants also with Putrescine and normalize the data?

Author’s response: As we mentioned that this is the first report of the potential beneficial roles of exogenous Put in modulating drought stress tolerance in sugar beet by regulating physio-biochemical traits and gene expression. Our main research purpose was to analyze the response of sugar beet seedlings by exogenous Put under drought conditions. Many studies already have reported that the exogenous application of Put in control plants showed a significant influence on the seedlings growth and various plant physiological and biochemical processes. That’s why we did not consider the Put treatment on control plants in the present experiment. However, we have treated the control plants with the same amount of water as we have used the amount of Put solution in the drought-stressed plants to normalize the water effect.

Furthermore, why authors have not showed any images of plants for example how sugar beet plants looks after 10 days of drought stress and how Put treatment recovers severity of drought phenotype?

Author’s response: In the revised manuscript, we added a schematic representation of the findings where we used the image of BSRI Sugar beet 2 genotype grown under control, drought, and put+ drought treatment conditions that also reflecting the better phenological appearance of the seedlings in response to Put application (Fig 7).

Introduction:
Line no. 67-68; reference error need to be checked.

Author’s response: The reference error has been resolved accordingly.

Spacing errors.

Author’s response: The manuscript has been revised carefully and spacing errors have been eliminated accordingly.

Material and methods:
Line no. 115 to 117; explain in detail for how long (time) seeds were sterilized.

Author’s response: The information related to surface sterilization of seeds has been written newly, including duration accordingly.

Line 121 to 125; This is the important information for readers, authors should mention whether Put was sprayed for every day/how many times per day?

Author’s response: This part of the materials and methods has been revised as “At thirty-day-old stage the seedlings were subjected to drought stress at 30% field capacity (FC) for ten days while the control plants were grown at 90% FC. After that, the seedlings under drought stress were treated with Put at 0.3 mM, 0.6 mM, and 0.9 mM concentration level, where control plants were treated with water only. Put, and water was applied to both sides of the leaves of seedlings for one time with 1% Tween-20 (v/v).”

Line 176: reference error, numbering not mentioned to reference.

Author’s response: The reference error has been resolved accordingly.

Result and Discussion:
Result section is hard to follow. Authors should consider to rewrite it.
Line 226 to 236: written very poorly, Authors should consider rewriting this section carefully, What is Kaveri? The table 2 is very confusing, there are no units to Shoot dry wt, root dry wt, leaf area, LRWC. Again, new Kaveri genotype which is not mentioned in materials and method section!

Author’s response: The Table 2 has been reformed and analyzed newly. The name of the variety has been corrected as BSRI Sugar beet 2. The units of all parameters in Table 2 have been added accordingly. The results section has been revised accordingly.

Plant growth and parameters: At which day samples were collected/analyzed not mentioned.

Author’s response: The information related to samples collection/ analysis has been added to the materials and methods section as “After ten days of treatment, the plant samples were randomly collected for further analysis.”(line 125)

At line 247 to 250, there is no flow to the sentence.

Author’s response: The discussion part of the plant growth parameters has been revised accordingly.

Line 302 to 306, there is no flow while reading, consider rewriting.

Author’s response: The discussion part of OJIP parameters has been revised accordingly.

Antioxidant Enzyme Activity:
Line 405 to 406: Written wrong! Antioxidant enzyme levels were reduced after drought in both genotypes but its written increased; though its being increased in Put treatment compared to drought; but this has been not mentioned. The results written in Antioxidant enzyme activity need to rewrite carefully.

Author’s response: Thank you for your very good observation. After a careful check, we revised the results and discussion part of antioxidant enzyme activity accordingly.

Relative gene expression:
If the relative gene expression is normalized to reference gene, then it should be mentioned and if it is compared to something then it is fold change or Log2fold change?
Authors need to consider checking the expression of Put biosynthesis genes namely, arginine decarboxylase (ADC) and Orn decarboxylase (ODC) in their sugar beet samples. Authors have checked expression of antioxidant genes after 10 days of drought, earlier time point is important. Moreover, whether does Put treatment to control sugar beet plants/genotypes alters the expression of these antioxidant genes has not been verified, short experiment would be to just treat sugar beet seedlings with Put for several timepoints and check expression of these antioxidant genes along with Put biosynthesis genes.

Author’s response: The relative gene expression levels were calculated using the following equation: △△Ct = △Ct, _treatments_ – △Ct, _calibrator_. where △Ct, _treatments_ is the Ct values for treatments (Drought &putrescine) cells normalized to Actin (Ct[target gene]_treatments_ – Ct[Actin]_treatments_) and △Ct, _calibrator_ is the Ct values for the control (untreated) cells normalized to Actin (Ct[target gene]_calibrator_ – Ct[Actin]_calibrator_). For the validation of the comparative Ct method (2^-△△Ct^), the amplification efficiencies of the target genes and reference gene (Actin) were evaluated. The following statement was not included in this manuscript because of general and prerequisite information (efficiency, sensitivity, and precision) for qPCR work. We, all authors, agree to the comment that checking the expression Put biosynthesis genes would be worthwhile, however this experiment was design to check roles of exogenous putrescine in related antioxidant genes expression and compered with one time point to keep consistency with other experiments. Put treatment at several time points and check expression of these antioxidant genes along with Put biosynthesis genes are our ongoing plan.

Figures:
Figure legends must be rewritten! In all figures, It is very confusing to determine the significant differences between the treatments, Authors should consider labeling significant differences by different letters and non-significance by same letter. This way it is easy for readers to follow. Moreover, Authors should explain in figure legend about the asterisk/ letters (if newly added) denotes significant differences between which treatment.

Author’s response: The legends have been written in all figures accordingly. Necessary explanations related to figure legends and asterisks have been added accordingly.

Reviewer #2: In the manuscript (ms) entitled “Exogenous putrescine attenuates the negative impact of drought stress by modulating physio-biochemical traits and gene expression in sugar beet (*Beta vulgaris* L.)” authors put quite a lot effort. Here they investigated the potential roles of exogenous putrescine (Put) in improving drought stress tolerance of two sugar beet genotypes by assessing morpho-physiological and biochemical traits, as well as gene expression.
In my opinion, the findings of this study would be pretty interesting and important for the readers from related field. However, the ms in the current state lacks cohesiveness in scientific writing and prepared carelessly in terms of writing norm. Based on the following observations the article in its current status is not suitable to publish in this journal.

Author’s response: Many thanks for your valuable comments and suggestions regarding our MS. We made substantial improvements to our MS based on your comments to make it more meaningful.

**Specific comments**

1. In the abstract, the authors mainly discussed the methods and listed the overall results instead of presenting the main findings. Authors are suggested to synthesize the main findings here highlighting special techniques/methods used.

Author’s response: Thanks for your critical comments. We have revised the abstract to make it more consequential.

2. The main drawback of this ms is poor presentation and explanation of results. In presenting comparative results, authors in most of the cases just have used the terms ‘significant’, ‘prominent increase’, ‘increased/decreased’. In this case, authors should focus on quantifying (where possible) the recovery of drought-induced losses in sugar beet in response to Put treatments. Additionally, authors should include a comparative phenotype figure on Put-induced drought tolerance in sugar beet to strengthen their claim.

Author’s response: Thank you for your valuable suggestions. We revised the manuscript, and necessary information especially quantifying the recovery of drought-induced losses, has been incorporated.

3. The subsequent discussion supporting the results has also been very superficial. For instance, in Line (L)-270, authors mentioned “these results have been confirmed in other studies”. How their results confirmed by others? Authors should clearly express what they meant here?

Author’s response: The above mentioned discussion part has been modified as “similar results were also described in *Panax ginseng* where 0.3 mM Put recovered the photosynthetic pigments of saline-stressed seedlings.”

4. The ms requires several rounds of English polishing. Specifically, there are lots of typos, long sentences and scientifically incorrect sentences in the ms, which might confuse the readers to get clear insights. Inconsistency in writing style (abbreviation, citation etc.) should also address very seriously.
Some specific concerns, but by no means an exhaustive list, mentioned below should be addressed to improve the current status of the ms:
-L67: Citation style is found inconsistent

Author’s response: The citation error has been resolved accordingly

-while discussing the results in L225: surprisingly, in table 2, no ‘'BSRI Sugar beet 2’ was found!! Here they mentioned the genotype name 'Kaveri' instead of 'BSRI Sugar beet 2'. It seems authors carelessly mentioned ‘Kaveri’ here!! Or, something else, please clarify.

Author’s response: We apologize for our mistake. The name of the variety has been corrected as BSRI Sugar beet 2 in the table and results section accordingly.

-L231-232: “In the case of SBT-010, although SDW and LA recovered
most under 0.6 mM and 0.9 mM Put, respectively, but no positive effect was observed for RDW”. As said earlier, from such types of ‘English’ readers might lose their interest. Please improve the English standard to have readers’ interest.

Author’s response: The results and discussion relate to growth parameters have been revised accordingly. The English of the MS is also re-checked by our co-authors from native English country.

L258: chlorophyll "a" and "b" should write in italic!

Author’s response: Chlorophyll "a" and "b" have been written in italic in the revised manuscript.

L280: Results explained on 'fluorescence parameters' is not understandable at all!

Author’s response: The results relate to fluorescence parameters have been revised accordingly.

L281: What is meant by OJIP? Author should elaborately mention when any abbreviation appears first.

Author’s response: The fluorescence induction kinetics is termed OJIP analysis. In the results section, ‘fluorescence induction kinetics’ has been used as the elaboration of OJIP.

L294-296: Unnecessary discussion.

Author’s response: After revised carefully, line no 294-296 of the discussion part has been deleted from the manuscript.

L405-411: Very hard to understand the activity of enzymes. This explanation is not even consistent with Fig. 4!

Author’s response: The results section of antioxidant enzyme activity has been revised accordingly.

L469: Authors mentioned ‘it was revealed that cluster-A was mostly characterized by…….’ They should avoid such kind of vague and non-scientific wording to present the data.

Author’s response: The discussion part related to hierarchical clustering has been revised accordingly.

L485-491: Authors should discuss the PCA results more elaborately to clearly focus the main traits contributed greatly to Put-mediated drought tolerance in sugar beet.

Author’s response: The PCA results have been revised accordingly.

L519-520: In the last line of conclusion “Importantly, Put at a medium dose (0.6 mM) was found to be most effective in modulating the morpho-physiological and biochemical processes in sugar beet plants under drought stress. I wonder how authors reached on a conclusion like this! Besides, the term ‘medium dose’ is a vague and non-scientific word!

Author’s response: The conclusions have been revised accordingly.

-the figure and table legends are not complete. Authors should give more care on writing it completely.
-in table 4: in presenting the Pro data of SBT-010, it seems authors mistakenly put the same letter ‘g’ for both ‘4.81 ± 0.48’ and ‘15.05 ± 0.07’. There must have significant difference between them! Authors should seriously address such kind of issue.

Author’s response: All the tables and figures have been checked and revised carefully. The legends have been written in all figures accordingly. Necessary explanations related to figure legends and asterisks have been added accordingly.

Further recommendation
It would be pretty interesting and worth reading for the related readers if authors could produce a flow/process diagram on Put-mediated drought tolerance mechanism in sugar beet based on the findings of the present study.

Author’s response: Thank you for your valuable suggestion. A diagram entitled “A Schematic representation of drought-induced growth inhibition and improvement of growth by exogenously Put treatment on BSRI Sugar beet 2” has been added as Figure 7 in the revised manuscript.
