# Supplementary material for: Exogenous putrescine attenuates the negative impact of drought stress by modulating physio-biochemical traits and gene expression in sugar beet (Beta vulgaris L.)
Source: PLoS One. 2022 Jan 7;17(1):e0262099. doi: 10.1371/journal.pone.0262099 (PMC8741020; doi:10.1371/journal.pone.0262099)
Supplement: Attachment — Submitted filename: Reviwer and editor comments.docx [file pone.0262099.s007.docx]

Reviewer 1: I have incorporated your suggestions into my revision. They were very helpful. Thank you.

Reviewer 2: I have incorporated your suggestions into my revision. They were very helpful. Thank you.

Academic editor: I have incorporated your suggestions into my revision. They were very helpful. Thank you.
